# GAMIBHEAR: whole-genome haplotype reconstruction from Genome Architecture Mapping data

**DOI:** 10.1093/bioinformatics/btab238

**Published:** 2021-04-08

**Authors:** Julia Markowski, Rieke Kempfer, Alexander Kukalev, Ibai Irastorza-Azcarate, Gesa Loof, Birte Kehr, Ana Pombo, Sven Rahmann, Roland F Schwarz

**Affiliations:** Berlin Institute for Medical Systems Biology, Max Delbrück Center for Molecular Medicine in the Helmholtz Association (MDC), 10115 Berlin, Germany; Humboldt-Universität zu Berlin, Department of Biology, 10099 Berlin, Germany; Berlin Institute for Medical Systems Biology, Max Delbrück Center for Molecular Medicine in the Helmholtz Association (MDC), 10115 Berlin, Germany; Humboldt-Universität zu Berlin, Department of Biology, 10099 Berlin, Germany; Berlin Institute for Medical Systems Biology, Max Delbrück Center for Molecular Medicine in the Helmholtz Association (MDC), 10115 Berlin, Germany; Berlin Institute for Medical Systems Biology, Max Delbrück Center for Molecular Medicine in the Helmholtz Association (MDC), 10115 Berlin, Germany; Berlin Institute for Medical Systems Biology, Max Delbrück Center for Molecular Medicine in the Helmholtz Association (MDC), 10115 Berlin, Germany; Humboldt-Universität zu Berlin, Department of Biology, 10099 Berlin, Germany; Berlin Institute of Health (BIH) at Charité–Universitätsmedizin Berlin, 10177 Berlin, Germany; Regensburg Center for Interventional Immunology (RCI), 93053 Regensburg, Germany; Universität Regensburg, 93053 Regensburg, Germany; Berlin Institute for Medical Systems Biology, Max Delbrück Center for Molecular Medicine in the Helmholtz Association (MDC), 10115 Berlin, Germany; Humboldt-Universität zu Berlin, Department of Biology, 10099 Berlin, Germany; Genome Informatics, Institute of Human Genetics, University Hospital Essen, University of Duisburg-Essen, 45122 Essen, Germany; Berlin Institute for Medical Systems Biology, Max Delbrück Center for Molecular Medicine in the Helmholtz Association (MDC), 10115 Berlin, Germany; Berlin Institute for the Foundations of Learning and Data (BIFOLD), 10623 Berlin, Germany

## Abstract

**Motivation:**

Genome Architecture Mapping (GAM) was recently introduced as a digestion- and ligation-free method to detect chromatin conformation. Orthogonal to existing approaches based on chromatin conformation capture (3C), GAM’s ability to capture both inter- and intra-chromosomal contacts from low amounts of input data makes it particularly well suited for allele-specific analyses in a clinical setting. Allele-specific analyses are powerful tools to investigate the effects of genetic variants on many cellular phenotypes including chromatin conformation, but require the haplotypes of the individuals under study to be known *a priori*. So far, however, no algorithm exists for haplotype reconstruction and phasing of genetic variants from GAM data, hindering the allele-specific analysis of chromatin contact points in non-model organisms or individuals with unknown haplotypes.

**Results:**

We present GAMIBHEAR, a tool for accurate haplotype reconstruction from GAM data. GAMIBHEAR aggregates allelic co-observation frequencies from GAM data and employs a GAM-specific probabilistic model of haplotype capture to optimize phasing accuracy. Using a hybrid mouse embryonic stem cell line with known haplotype structure as a benchmark dataset, we assess correctness and completeness of the reconstructed haplotypes, and demonstrate the power of GAMIBHEAR to infer accurate genome-wide haplotypes from GAM data.

**Availability and implementation:**

GAMIBHEAR is available as an R package under the open-source GPL-2 license at https://bitbucket.org/schwarzlab/gamibhear.

**Supplementary information:**

Supplementary data are available at *Bioinformatics* online.

## 1 Introduction

Genome Architecture Mapping (GAM) is a novel digestion- and ligation-free experimental technique for assessing the 3D chromatin structure from sequencing a collection of thin cryosectioned nuclear profiles (NuPs) (Beagrie *et al.*, 2017). Chromatin contacts between DNA loci can be inferred from the frequency at which loci are captured in the same NuP. One advantage of GAM over competing methods, such as Hi-C (Lieberman-Aiden *et al.*, 2009), is that GAM only requires several hundreds of cells to obtain high-resolution contact maps (Beagrie *et al.*, 2020; Fiorillo *et al.*, 2020; Kempfer and Pombo, 2019). This makes GAM particularly useful for the study of chromatin contacts in rare biological materials, such as human biopsies. Recently, there has been increasing interest in the allele-specific analysis of chromatin contacts, for which haplotyping, i.e. phasing of single nucleotide variants (SNVs) is key (Cavalli *et al.*, 2019; Rivera-Mulia *et al.*, 2018), but so far no algorithm exists for haplotype reconstruction from GAM data.

*De novo* phasing is traditionally achieved through read-based methods such as HapCut, WhatsHap or HapCHAT (Bansal and Bafna, 2008; Beretta *et al.*, 2018; Edge *et al.*, 2017; Patterson *et al.*, 2015). In these methods, variants of the Minimum Error Correction (MEC) problem are used with varying error distributions and insert lengths (Bansal and Bafna, 2008). MEC views the given data (a fragments by SNV sites matrix of observed allele states) as potentially erroneous and asks for the least invasive way to correct the observations to enable conflict-free phasing. The MEC problem is computationally hard under a variety of conditions (Bafna *et al.*, 2005; Cilibrasi *et al.*, 2005). As a heuristic, HapCut converts MEC to a maximum cut problem and originally allowed for only single base-pair errors (Bansal and Bafna, 2008). Selvaraj *et al.* (2013) later leveraged chromosome territories (Meaburn and Misteli, 2007) and extended HapCut to Hi-C data by accommodating Hi-C specific h-trans errors. H-trans errors are haplotype switch errors that occur when a genomic region interacts with another genomic region located on the other homologous chromosomal copy (in trans). HapCut2 now includes population-based statistical phasing (Bansal, 2019) and implements a variety of different error models to accommodate different sequencing technologies (Edge *et al.*, 2017).

Alternative formulations to the phasing problem seek to partition the observed fragments (Duitama *et al.*, 2010), or the aggregated co-occurrence frequencies of SNVs (Tourdot and Zhang, 2019), into two classes corresponding to the two haplotypes by minimizing a measure of inconsistency. To facilitate haplotype reconstruction from GAM data, we here also use an aggregation step and formulate the problem on co-occurrence evidence derived from the raw GAM NuPs. This formulation is equivalent to finding a ground state to the well-known spin glass system from physics, which is equivalent to a maximum cut problem (Tourdot and Zhang, 2019).

The resulting algorithm GAMIBHEAR (GAM-Incidence Based Haplotype Estimation And Reconstruction) employs a graph representation of the co-occurence of SNV alleles in NuPs for whole-genome phasing of genetic variants from GAM data. GAMIBHEAR accounts for the GAM-specific probabilities in capturing parental chromosomal segments as part of the random cryosectioning process. We assess the performance of GAMIBHEAR on the hybrid mouse embryonic stem cell line F123 with known haplotype structure. Despite the sparsity of GAM data, GAMIBHEAR allows for accurate, dense, genome-wide haplotype reconstruction. GAMIBHEAR is available as an efficient R package with parallel implementations of the most compute-intensive tasks and is available at https://bitbucket.org/schwarzlab/gamibhear.

## 2 Materials and methods

### 2.1 Definitions, problem statement and objective

Our goal is to reconstruct haplotypes from GAM data. A sequenced GAM dataset consists of reads from many nuclear profiles (NuPs). Each NuP is the result of random sectioning of the nucleus and captures ultra-sparse *local* sequence information, where *local* refers to genomic loci in close proximity in the 3D arrangement of the genome, including but not limited to loci proximal in linear distance. Thus, reads from single NuPs cover a small proportion of the whole genome with consecutive stretches of genomic DNA that reflect chromatin looping in and out of a thin nuclear slice (illustrated in Fig. 2B). Our main assumption here is that alleles of any two heterozygous SNVs captured in a nuclear slice are likely to originate from the same parental copy, and that this likelihood decreases with increasing genomic distance between the two SNVs.

We assume that the set of heterozygous SNVs is given and that the SNV alleles have been determined per NuP. Let N be the number of NuPs and M be the number of heterozygous SNVs in the genomic region of interest (e.g. a chromosome or chromosome arm; sites with homozygous SNVs are ignored). Then the problem input is a ternary N×M matrix D with $D_{ij}=1$ if the reference allele is observed in NuP i at SNV site j, $D_{ij}=-1$ if the alternative allele is observed, and $D_{ij}=0\;$if there is no unique observation (e.g. due to lack of coverage or if both alleles are observed in the same NuP).

The goal is to reconstruct the two haplotypes (allele states on the same parental copy). Formally, a haplotype is a vector $h\in \{-1,1{\}}^{M}$ with $h_{j}=1$ if the reference allele is found at site j and $h_{j}=-1$ for the alternative allele. One of the two haplotypes h determines the other one as -h.

The GAM input data in principle contains the information to infer h. Consider the relation between SNV sites j and k in NuP i. The two sites can be in a ‘flip’ relation, where the alternative (alt) allele (-1) of one site is observed with the reference allele (+1) of the other site (product $D_{ij}\cdot D_{ik}=-1$), and a ‘stay’ relation, where both SNVs show either the reference or alternative allele (product $D_{ij}\cdot D_{ik}=1$).

We thus compute the M×M  *evidence matrix* $A:=D^{T}D,\;$which contains the accumulated counts of the stay-flip relations summed over all NuPs, i.e. $A_{jk}=\sum_{i=1}^{N}D_{ij}\cdot D_{ik}$, such that positive values indicate more stay observations ($A_{jk}>0$: ‘stay’ between sites j and k; j,k=1,…, M) and negative values indicate more flip observations ($A_{jk}<0$: ‘flip’ between sites j and k). An equal number of observed stays and flips leads to zero entries ($A_{jk}=0$).

The goal of the haplotype reconstruction algorithms we develop here is to solve h using the information contained in A: If $A_{jk}>0$, then we should have $h_{j}=h_{k}$, and if $A_{jk}<0$, then $h_{j}=-h_{k}$. However, the information in A may be conflicting when considering transitivity: Consider three sites j,k,l with $A_{jk}>0,\;A_{kl}>0,\;A_{jl}<0$. Thus, decisions need to be made on how to resolve conflicting information in the evidence matrix A.

We formulate the problem as follows: Given the M×M matrix A, we seek $h\in \{-1,1{\}}^{M}\;$to maximize $F(h):=\sum_{j<k}h_{j}A_{jk}h_{k}.$

This formulation encourages $h_{j}$ and $h_{k}\;$to take the same sign if $A_{jk}>0$ and different signs if $A_{jk}<0$. This maximization problem is equivalent to finding an exact ground state for a spin glass in physics and is known to be NP-hard in general and can be cast as a maximum cut problem on a graph induced by A (Tourdot and Zhang, 2019). Here we propose heuristic algorithms that make use of known properties of the evidence matrix A (potentially proximity-scaled; see below) and evaluate them against a dataset with a known correct solution.

Before we state two such algorithms, let us first relax our notion of what we accept as a solution. Above, we defined a (fully resolved) haplotype as a vector $h\in \{-1,1{\}}^{M}$ with $h_{j}=1$ if the reference allele is found at site j and $h_{j}=-1$for the alternative allele. However, the available data may not be sufficient to fully resolve the haplotype. Where no phasing information is available, we allow partial solutions (‘blocks’) as follows. Let ${J:\;=\;(J}_{1},\;J_{2},\;\ldots ,\;J_{K})$ be a partition (disjoint union) of {1,…,M} into K blocks. Then a solution of the GAM haplotype reconstruction problem for input matrix D with partition J is a collection of K binary vectors $h^{1}\in \{-1,1{\}}^{J_{1}},\;\ldots ,\;h^{K}\in \{-1,1{\}}^{J_{K}}$. Each of the K blocks is solved independently, and no statement is made about the connection between these blocks. The blocks are often intervals, but may be arbitrary subsets of all sites, especially for GAM data. Obviously, solutions with fewer independent blocks are more desirable.

### 2.2 Haplotype reconstruction algorithms

#### 2.2.1 Neighbour phasing

We first consider a baseline phasing strategy that leverages the most reliable short-range haplotype information on neighbouring SNVs only (‘neighbour phasing’). In the above notation, we only consider the first off-diagonal of A, i.e. $A_{j,(j+1)}$ for j=1,…,M. Essentially, this resolves possible conflicting information by ignoring a large fraction of the available data, and only considering a single path between any two sites j≤k: j→j+1→⋯→k. The reconstructed haplotype starts (arbitrarily) with the reference allele, thus $h_{1}=1$. Once $h_{j}$ is determined, we set $h_{j+1}:=h_{j}\cdot$sign($A_{j,(,j+1)}$), i.e. we stay or flip according to the sign of $A_{j,(j+1)}$. In case of a tie or when SNV j and j+1 are never co-observed in the same NuP ($A_{j,(j+1)}=0$), we start a new independent block where $h_{j+1}=1$. Solutions produced by neighbour phasing consist of blocks that are intervals. The resolved blocks can be expected to be correct with high probability, but also short, and therefore of limited use.

#### 2.2.2 Graph phasing with optional proximity scaling

**Fig. 1.**
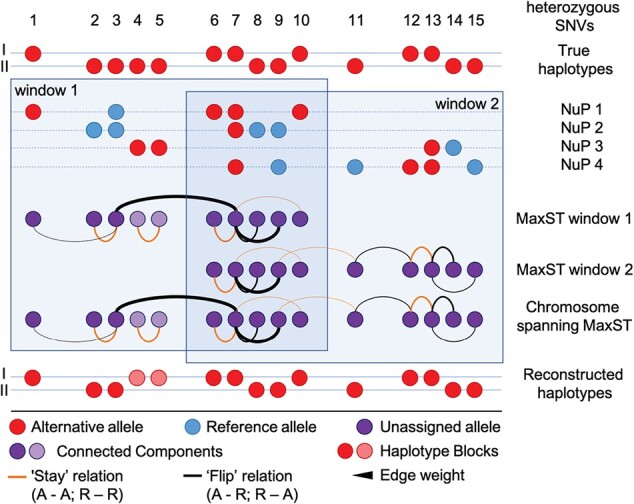
Schematic overview of the graph phasing algorithm. The location of alternative alleles of heterozygous SNVs on the two parental chromosomes describes the true haplotypes (top). NuPs 1–4 are sparse local samples of the true haplotype structure. At heterozygous SNV positions, either the alternative (red) or reference allele (blue) can be observed. In overlapping windows, graphs of co-observed SNVs are built over all NuPs. Edges are of either stay (orange) or flip (black) type and edge weights correspond to the co-observation frequency (line width) and are optionally proximity-scaled. A set of SNVs that is itself not co-observed with other SNVs in the same window forms its own connected component in the graph (e.g. SNV 4 and SNV 5 in NuP 3, window 1). MaxSTs (forests in case of multiple connected components) are calculated per window and combined to yield a sparse but chromosome-spanning graph. The MaxST of this sparse graph is used to assign alternative alleles to the final reconstructed haplotypes (bottom). Connected components in the final MaxST form separate, possibly nested haplotype blocks (red/pink). As the leftmost SNV of each separate haplotype block is assigned to haplotype 1, SNVs 4 and 5 are correctly phased relative to each other (stay relation), but assigned to the wrong haplotype.

We extend the local proximity of SNVs from immediate neighbours to larger genomic windows using a graph-based approach (Fig. 1). To improve computational efficiency each chromosome is divided into windows of a fixed number L of SNV sites with half a window size overlap. Phasing is carried out on each window independently and results per window are subsequently reconciled (see below). To process a window, we restrict the N×M input matrix $D=(D_{ij})$ to the window's sites and only consider the reduced N×L matrix D and the derived L×L evidence matrix ${A=(A}_{jk})$. We systematically evaluated different windows sizes in terms of runtime, memory usage and phasing completeness and accuracy. We settled on L = 20 000 SNVs as the default, as it causes only a marginal reduction in accuracy while improving completeness and drastically reducing computational demands (see Supplementary Note S6).

As we assume that the reliability of phasing information within a NuP decreases with genomic distance, we include an option to scale the information in A element-wise by a weight matrix $W=(W_{jk})$, where $W_{jk}\;$depends on the genomic distance $d_{jk}$ between sites j and k. We use a simple exponential decay model, where $W_{jk}=C\cdot \;\text{exp}(-\lambda \;d_{jk})$ for $d_{jk}$ in a certain range $\left[D_{\min},D_{\max}\right]$, and $W_{jk}=1$ for $d_{jk}<D_{\min}$ and $W_{jk}=0$ for $d_{jk}>D_{\max}$. The choice of appropriate parameters C>0, λ>0 and ${0\leq D}_{\min}<D_{\max}$ is discussed below. In the following, A represents the *proximity-scaled evidence matrix* ($A_{jk}\leftarrow W_{jk}\cdot A_{jk}$).

At this point, there are four potential reasons for $A_{jk}=0$: First, sites j and k may never co-occur in any NuP. Second, they may never be considered in the same window of L sites. Third, their genomic distance may be larger than $D_{\max}$. Fourth, an equal number of observations of stay and flip relations may be encountered between sites j and k.

The non-zero entries in A induce an undirected weighted graph. Its L vertices are the sites of the current window. An edge between sites j and k exists with weight $A_{jk}$ if $A_{jk}\;\neq \;0$. Two sites in the same connected component of this graph are typically connected by many paths. Consider a single arbitrary path between sites j and k. The number of negative-weighted edges along the path determines the haplotype assignment: if the number is even, then $h_{k}=h_{j};\;$if it is odd, then $h_{k}={-h}_{j}.$ Different paths between the two sites can be conflicting in their haplotype assignment. However, if the graph is reduced to a tree (or forest in case of more than one connected component), there is a unique path between each pair of sites (in the same connected component). Because the absolute values $\left|A_{jk}\right|$ indicate strength of direct evidence for the relation between sites j and k, we compute a *maximum spanning tree* (MaxST) of each connected component based on absolute edge weights $\left|A_{jk}\right|$ using Kruskal's algorithm. Recall that the problem is solved on (potentially dense graphs of) windows, so the required running time is $O(L^{2}l\mathrm{ogL})$ for each window. The MaxST approach has the property that the resulting path between any two sites j and k maximizes the minimum weight of the path's edges among all possible paths between j and k (Hu, 1961), so we construct the graph by maximizing the weakest evidence link between each pair of sites of the window, which appears to be a reasonable heuristic for the given problem. The computed MaxST then determines the haplotypes (or set of haplotype blocks in case of a forest of MaxSTs) for the current window.

To infer haplotypes across the whole chromosome, the MaxSTs of overlapping windows must then be joined into a chromosome-wide graph. For this, we join the (overlapping) MaxSTs of all windows into a new graph consisting of all M SNV sites as nodes and the union of edges of all MaxSTs. Because each node is in at most two MaxSTs, the number of edges in the union is bounded by 2(M-1). In order to solve possible disagreements stemming from the results of overlapping windows in this sparse graph, we again determine a MaxST (if necessary, on each connected component separately) in O(M logM) time to obtain a unique path between any two connected sites.

For the output, each connected component defines an independent block. The haplotype of the leftmost SNV site $h_{1}$ (with smallest genomic coordinate) in each block is arbitrarily set to $h_{1}:=1$, and the other states $h_{j}$ are computed according to the number of negative-weighted edges on the unique MaxST path between the first site and j.

Including phasing information from non-adjacent SNV pairs will improve completeness and yield larger, potentially chromosome-spanning haplotype blocks. In the reconstructed haplotypes of the graph phasing approach, blocks can be nested. The inclusion of phasing information from more distant SNV pairs might compromise the overall accuracy of the results, however, the proximity scaling is expected to keep the introduction of misleading information to a minimum.

### 2.3 Performance measures

To assess the quality of the reconstructed haplotypes we compare GAMIBHEAR estimates with the haplotypes of the F123 mouse embryonic stem cell (mESC) line obtained from whole-genome sequencing of the parental mouse strains (see Supplementary Note S1). The overall quality of reconstructed haplotypes depends on both the completeness of the reconstructed haplotype blocks as well as the phasing accuracy of the SNVs contained.

In terms of completeness, we report the total proportion of phased heterozygous SNVs next to the standard S50 (Lo *et al.*, 2011), N50 (Lander *et al.*, 2001) and adjusted N50 (AN50; Lo *et al.*, 2011) metrics which give an impression of the median size (in SNVs) and span (in bp) of the reconstructed haplotype blocks. To enable comparisons with previous phasing approaches of the F123 cell line (Selvaraj *et al.*, 2013) we report the metrics in percent of the phasable variants (number of input variants) and phasable genome (range between leftmost SNV and rightmost SNV per chromosome, 97.58% of the genome), respectively.

To evaluate accuracy, we report the Switch Error Rate (SER), defined as the proportion of adjacent variant pairs that were phased incorrectly out of all phased variant pairs. We also report the adjusted Switch Error Rate (adjusted SER) to account for incomplete or fragmented phasing results, by penalizing unresolved transitions between adjacent variant pairs with 0.5 switch errors, to account for, on average, a 50% chance of assigning the wrong phase. Fragmented phasing occurs when the phasing graph is composed of many small components with phasing information within but not between components. Additionally, the global haplotype agreement is reported, calculated by direct comparison of the reconstructed and true haplotypes (i.e. alt-ref configurations). To be able to relate the results to the size of the GAM dataset, we also report the quality of haplotypes reconstructed from incrementally increasing subsets of 100 NuPs chosen at random in ten iterations (Fig. 3). All performance measures are given in averages across all 19 chromosomes. For a more detailed description and motivation of the individual metrics please see Supplementary Note S4.

### 2.4 GAMIBHEAR implementation

The presented haplotype reconstruction algorithms are implemented in the R package GAMIBHEAR. GAMIBHEAR is open source and freely available under the GPL-2 license at https://bitbucket.org/schwarzlab/gamibhear.

## 3 Results

### 3.1 Benchmark dataset

The F123 mouse embryonic stem cell line was derived from a hybrid F1 mouse resulting from the cross of the two inbred, homozygous mouse strains *CAST (Mus musculus castaneus)* and *J129 (Mus musculus domesticus J129)*. The F1 generation is thus heterozygous at all loci for which their parents have different alleles. As the parental mouse strains are both fully sequenced, the haplotypes of the F123 cell line were derived from SNV sets called on the parental strains (see Supplementary Note S1). Its known haplotype makes the F123 cell line an ideal model for benchmarking phasing algorithms.

**Fig. 2.**
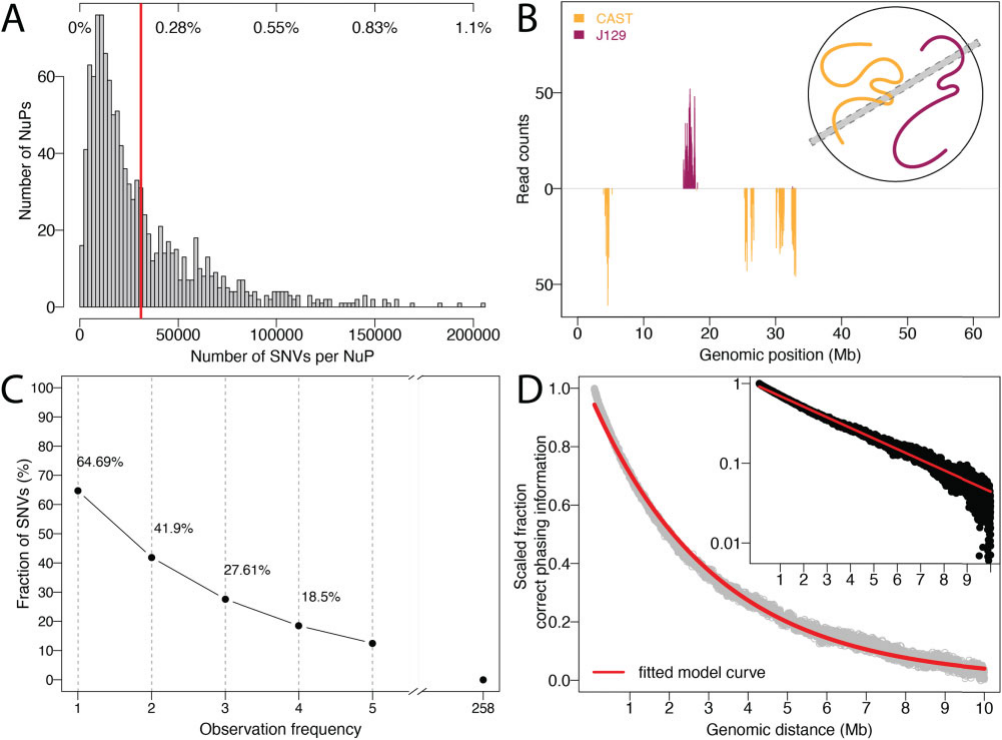
GAM captures local phasing information. (**A**) Histogram of the number of observed SNVs per NuP in the F123 dataset (fraction of all SNVs at top, mean = 0.171%, red line). (**B**) Example of read counts supporting the CAST (orange, downwards) and J129 (red, upwards) alleles in a single NuP on chromosome 19, visualizing the sparsity of GAM data. Inset depicts physical capturing of respective genomic regions in a slice (grey area) by cryosectioning in a GAM experiment. (**C**) Cumulative fraction of SNV observation frequencies. 64.69% of SNVs are observed at least once, 41.9% of SNVs are observed at least twice across all NuPs. (**D**) The fraction of correct phasing information decreases exponentially with increasing genomic distance of observed SNV pairs. The fit of the exponential curve to the fraction of correct phasing information of SNV pairs with genomic distance between 1 bp and 10 Mb is shown in red. The inset shows the decrease of correct phasing information on a logarithmic scale.

Using the novel GAM method, 1281 single NuPs were generated from the F123 mESC cell line (available at 4D Nucleome Consortium data portal accession number 4DNBSTO156AZ), out of which 1123 passed quality control (unique 4DN identifiers provided in Supplementary Data). We extracted on average 305 377 reads from 1123 NuPs, covering 0.171% (±0.167) of the 18 150 228 heterozygous SNVs per nuclear slice (Fig. 2A); exemplary data of genomic regions captured in a single NuP is shown in Figure 2B. Out of all F123 SNVs, 11 741 055 (64.69%) were observed at least once, 7 605 321 SNVs (41.9%) were observed at least twice (Fig. 2C).

For more details on F123, the generation of the benchmark haplotypes and the data preprocessing see Supplementary Notes S1–S3.

### 3.2 Exponential proximity scaling

Our method includes the option of exponentially downweighting evidence information $A_{jk}$ with increasing genomic distance (see Section 2.2.2). To validate this assumption and to choose optimal decay parameters, we examined the empirical probability p of two alleles coming from the same haplotype in the F123 data based on their genomic distance d and fit an exponential function $p=C\cdot e^{-\lambda \cdot (d-D_{\min})}$ using non-linear least squares. For this model we only considered pairs of sites within the interval $\left[D_{\min},D_{\max}\right]$= [1 bp, 10 Mb], where the decay in phasing information is most pronounced (Fig. 2D). The distance can be individually assigned by the user and probabilities 1 and 0 are assumed below $D_{\min}$ and above $D_{\max}$ respectively. Parameter C=1 then describes the co-observation probability at a genomic distance of 1 bp with an exponential decay parameter of $\lambda =3.173\cdot 10^{-7}$. The simple exponential dependency well describes the empirical distribution (Fig. 2D) and thus appears to be a good model for the reliability of the raw evidence as a function of genomic distance. In the following, we evaluate our graph phasing approach with and without proximity scaling.

### 3.3 Performance of GAMIBHEAR

#### 3.3.1 High quality haplotype reconstruction from GAM data

We evaluated the quality of the haplotypes reconstructed with GAMIBHEAR in terms of completeness and accuracy by comparing results to the true haplotypes of the F123 cell line (Table 1).

**Table 1.** Comparison of quality measures for the neighbour phasing algorithm, basic and proximity-scaled graph phasing algorithm for the full dataset.

	Neighbour phasing	Graph phasing (basic)	Graph phasing (proximity-scaled)
% phased SNVs	95.94% (±0.25)	99.97% (±0.004)	
% phased transitions	83.02% (±0.575)	99.96% (±0.00602)	
S50 absolute	10.84 SNVs (±0.5)	617 561.5 SNVs (±149 018)	
S50 percent	0.00188% (±0.00062)	99.94% (±0.010)	
N50 absolute	741.74 bp (±40.54)	126 454 374 bp (±32 645 641)	
N50 percent	0.00063% (±0.00021)	> 99.99% (±0.00003)	
AN50 absolute	741.74 bp (±40.54)	126 374 367 bp (±32 623 080)	
AN50 percent	0.00063% (±0.00021)	99.94% (±0.010)	
Global accuracy	85.87% (±3.53)	95.13% (±0.57)	94.28% (±8.45)
SER	0.76% (±0.13)	5.42% (±0.50)	2.09% (±0.26)
Adjusted SER	6.61% (±0.18)	5.43% (±0.50)	2.10% (±0.26)

*Note*: The mean of per-chromosome values is reported, standard deviation in brackets. Percent phased SNVs and transitions are reported in relation to observed SNVs. For a per chromosome report of accuracy results see Supplementary Note S5.

**Fig. 3.**
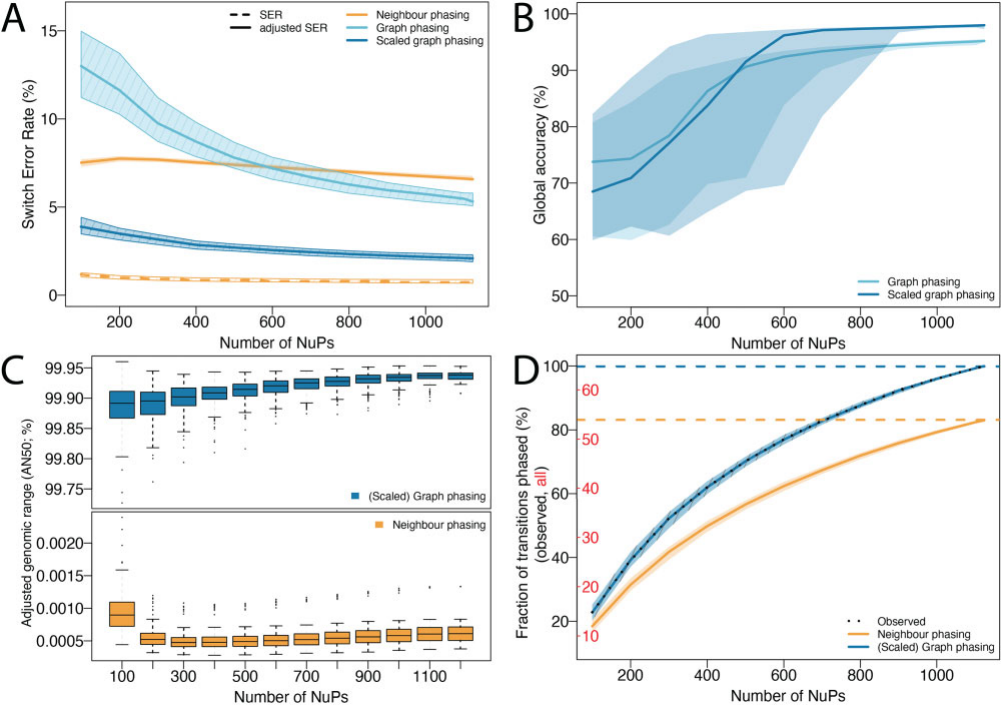
Quality of reconstructed haplotypes after neighbour phasing (orange), basic graph phasing (light blue) and proximity-scaled graph phasing (blue) for an increasing number of NuPs. Lines show the median value, shaded areas indicate the interquartile range of results across all chromosomes. (**A**) Local accuracy (SER): in graph phasing, SER decreases with an increasing number of NuPs as more information becomes available. Neighbour phasing in contrast shows a low SER independent of sample size (dashed orange line) due to a small number of phased transitions which are accurate. Adjusted SER penalizes unphased transitions and shows this difference: neighbour phasing performance (solid orange line) is substantially lower, graph phasing performance is unchanged (SER and adjusted SER lines overlap). Proximity-scaled graph phasing shows lowest adjusted SER overall. (**B**) Global Accuracy (haplotype agreement) improves with increasing sample size and proximity scaling further improves performance. (**C**) Completeness (AN50): graph phasing reconstructs dense, nested chromosome-spanning blocks even for low sample sizes (top), independent of proximity scaling. Neighbour phasing yields a large amount of small unconnected adjacent blocks, which are never nested, thus N50 = AN50 (bottom). (**D**) Completeness (% transitions phased): percentage of transitions phased relative to all known SNVs (red) and all SNVs observed at least once in the full dataset (black, see Fig. 2C). The number of observed SNVs and thus phasable transitions increases with increasing number of NuPs (dashed black line). Graph phasing predicts 99.96%, neighbour phasing predicts 83.02% of observed transitions.

**Neighbour phasing performance.** The neighbour phasing algorithm was built to exploit the most reliable short-range haplotype information of neighbouring co-observed SNVs, at the expense of completeness. This conservative algorithm shows the lowest switch error rate (SER) of the reconstructed haplotypes (0.76%, Fig. 3A), demonstrating strong local phasing information in GAM data. However, although over 95% of input SNVs were phased into adjacent haplotype blocks of at least size 2, the number of independent blocks is high (on average 79 965 blocks per chromosome), their size is small (Fig. 3C) and thus only 83% of possible transitions between neighbouring SNVs could be phased (Fig. 3D). Median haplotype blocks connect less than 11 SNVs (S50, 0.00188% of the phasable SNVs) and span less than 742 bp (N50, 0.00063% of the phasable chromosome), showing drastically low completeness. This low completeness is evident in the stark contrast between SER (0.76%) and adjusted SER (6.61%), confirming that neighbour-phasing yields small locally constrained but accurate phasing blocks (Fig. 3A). These locally accurate haplotypes confirm the presence of a strong local phasing signal in GAM data, but do not yield accurate phasing genome-wide. This algorithm shows the lowest global haplotype accuracy of 85.87%.

**Graph phasing performance.** The additional higher-order phasing information considered by the graph phasing algorithm substantially improves the completeness of the reconstructed haplotypes independent of proximity scaling (Fig. 3C). Over 99.9% of input SNVs were phased into haplotype blocks, over 99.9% of them into one main haplotype block (S50), spanning more than 99.99% of the phasable genome (N50) and phasing 99.96% of transitions (Fig. 3D). Adjusting the span of the largest block by the fraction of SNVs phased within yields an AN50 value of over 99.9% (Fig. 3C). The graph phasing algorithm thus reconstructs dense chromosome-spanning haplotypes.

Considering larger SNV windows increases the risk of integrating incorrect phasing information from co-observed SNV pairs located on homologous chromosome copies. Consequently, the accuracy of reconstructed haplotypes is lower compared to strict neighbour phasing. The basic graph phasing approach yielded results with ∼5% SER (Fig. 3A) and over 95% global accuracy (Fig. 3B). To improve accuracy while maintaining completeness we introduced proximity scaling and successfully reduced SER to ∼2% and increased global accuracy to ca 98% (Fig. 3A and B) with the exception of a few outliers (Supplementary Fig. S2). Those outliers are caused by a single switch error occurring within a haplotype block, which inverts the assignment of subsequent alleles, formally reducing global accuracy while maintaining SER and high, reliable local accuracy. Since the graph phasing resulted in highly complete haplotypes with a very low number of haplotypes blocks (on average 76 blocks per chromosome), the SER adjusted for unphased transitions only showed negligible changes compared to the unadjusted SER (adjusted SER: unscaled: 5.43%, scaled: 2.10%).

In conclusion, proximity-scaled graph phasing shows best performance overall and reconstructs accurate, chromosome-spanning haplotypes.

#### 3.3.2 Performance at lower SNV density

To show the effect of SNV density on the quality of haplotype reconstructions, we subsampled the F123 SNV set ( ∼8 SNVs per 1 kb) to resemble human SNV density ( ∼1–1.5 SNVs per 1 kb, 1000 Genomes Project Consortium *et al.*, 2015) and evaluated the resulting haplotypes reconstructed using the best-performing proximity-scaled graph phasing algorithm (see Supplementary Note S7).

GAMIBHEAR reconstructed accurate, dense, chromosome-spanning haplotypes: 99.96% of input SNVs were phased, of which 99.95% are within the main, chromosome-spanning haplotype block. This block spans 100% of the phasable genome (97.56% of the full genome). The median global accuracy of 96.64% and the switch error rate of 4.84% show that the quality of the reconstructed haplotypes in a subsampled dataset is only slightly lower compared to the haplotypes reconstructed from the full dataset, indicating that the algorithmic approach is largely independent of SNV density and thus applicable to human data. GAMIBHEAR thereby showed greatly improved resolution at a slightly reduced global accuracy compared to HaploSeq on comparably downsampled data ( ∼32% of input SNVs phased; 98.9% global accuracy) (Selvaraj *et al.*, 2013) (Supplementary Note S9).

#### 3.3.3 Time and memory usage

Phasing 11 741 055 heterozygous variants from the full 1123 NuP GAM dataset took approximately 1.5 h and 16 GB (largest chromosome 1: ca. 14 min, 0.9 GB) using the neighbour phasing algorithm, ca. 5 h and 30 GB using the basic/proximity-scaled graph phasing algorithm (largest chromosome 1: ca. 27 min, 26 GB) with default settings on a desktop PC with 64 GB of RAM without parallelization. However, computation can be carried out in parallel on multiple chromosomes for a further speed increase using the ‘cores’ option. Reconstructing haplotypes from the dataset subsampled to human SNV density using the best performing proximity-scaled graph phasing algorithm took 38 min and 20 GB for the whole genome (largest chromosome 1: ca. 3 min, 12 GB). For more details see Supplementary Note S6.

### 3.4 Comparison with existing methods

We compare GAMIBHEAR to the haplotype assembly methods WhatsHap (wMEC solver) (Patterson *et al.*, 2015) and HapCHAT (k-constrained MEC solver) (Beretta *et al.*, 2018), both designed for reconstructing haplotypes from long reads. For this, we converted GAM NuPs into pseudo-long reads by adapting the ternary input matrix D (see Section 2.2 and Supplementary Note S8). WhatsHap has a maximum coverage threshold of 23 reads which is exceeded in the F123 GAM data on a small number (0.0073%) of SNVs. This resulted in the read selection heuristic of WhatsHap to select only 69 of 1087 pseudo long reads (6.35%), thereby retaining only 11 039 SNVs (1.17% of input SNVs). In conclusion, coverage constraints in WhatsHap prevent its direct application to GAM data. Recently, HapCHAT was introduced to address this shortcoming by merging reads that are likely to originate from the same chromosome copy before read selection. In HapCHAT 1087 pseudo long reads were thus merged into 691 reads, 63 of which were selected for subsequent phasing, covering 604 358 SNVs (64.18% of input SNVs). From these, HapCHAT reconstructed a chromosome spanning haplotype block, with a global accuracy of 81.36% and an SER of 11.38% (compared to a global accuracy of 98.03% and SER of 1.98% using GAMIBHEAR). The MEC cost was reported as 307 734. This shows that in addition to the differences in coverage, the unique properties of GAM data prevent direct application of long-read MEC solvers for phasing. For details see Supplementary Note S8.

## 4 Discussion

The phasing problem has been extensively studied and approaches to solve it are typically specific to and optimized for certain experimental designs and datatypes, such as Hi-C (Edge *et al.*, 2017) and long reads (Beretta *et al.*, 2018; Patterson *et al.*, 2015). Although both GAM and Hi-C capture the spatial proximity of SNVs in the nucleus, the coverage and error distributions of the GAM cryosectioning process are sufficiently different from those of Hi-C that existing MEC solvers are not directly applicable. In Hi-C, phasing information is contained in ligated chimeric reads of genomic loci harbouring at least two SNVs, which can be very distant in linear genomic space but typically from the same chromosomal haplotype. In contrast, in GAM, phasing information is contained in NuPs, which yield individual short reads of both haplotypes and only maintain haplotype fidelity locally. Thus, in contrast to Hi-C, where h-trans errors remain rare, GAM NuPs frequently switch haplotypes. A Hi-C dataset furthermore consists of millions of reads, of which only a small percentage is useful for phasing as they rarely cover two SNVs or more (Giorgetti *et al.*, 2016). In contrast, a GAM experiment has in the order of 10^3^ NuPs, but a GAM NuP covers many SNVs (Fig. 2B). A single NuP therefore contains many long stretches of haplotype-resolved SNVs that allow ‘neighbour phasing’, which is not available with Hi-C and which shows that phasing with Hi-C and GAM data are two distinct computational problems.

In addition, SNV coverage in GAM data varies greatly and non-uniformly, which interferes with MEC solvers for long-read data that are fixed parameter tractable in the coverage and thus require the maximum coverage per SNV to be low (Patterson *et al.*, 2015). To ascertain these differences, we tested GAM data on the long-read MEC solvers WhatsHap and HapCHAT. HapCHAT only yielded SERs > 10%, owing to differences in the underlying technologies: long reads are not affected by haplotype switches but will frequently include single-nucleotide sequencing errors; GAM data, however, shows frequent switches in observed haplotypes, affecting all following SNVs. Due to these fundamentally different data characteristics, MEC solvers designed for haplotype assembly from long reads yield unsatisfying results when employed on GAM data. We did not attempt to transform GAM data for use with HapCut2, as it has been well known and stated by the authors that the performance of HapCut2 strongly depends on the correct error model being used and no such model exists for GAM data (Edge *et al.*, 2017).

The closest comparable dataset was provided by Selvaraj *et al.* (2013), who reconstructed F123 haplotypes using HaploSeq, combining Hi-C data with the HapCUT phasing algorithm. The largest chromosome-spanning blocks from GAMIBHEAR and HaploSeq both span over 99.99% of the phasable genome. The largest block from GAMIBHEAR includes >99.9% of observed variants compared to about 95% of observed variants using HaploSeq, a slight improvement due to the large genomic span covered by GAM NuPs. When downsampling the F123 SNV set to human SNV density, HaploSeq and GAMIBHEAR are still able to generate chromosome-spanning, accurate haplotype blocks, however, only 32% of SNVs are phased in the largest block by HaploSeq, while 99.95% of phased SNVs are contained in the largest haplotype block by GAMIBHEAR (Supplementary Note S9).

Although GAMIBHEAR shows high completeness given its input data even at low coverage, the sparsity of the GAM data itself hinders overall completeness. While in the Hi-C data of Selvaraj *et al.* (2013) 99.6% of variants were covered by at least one read, in the GAM dataset only 64.69% of variants are captured. While the sparsity of GAM data does not challenge the generation of accurate 3D chromatin contact maps (Beagrie *et al.*, 2017), advances in the GAM experimental protocol might overcome this drawback in the future to improve phasing results. Additionally, incorporation of statistical phasing could expand the reconstructed haplotypes to uncovered SNVs.

Our proximity scaling model improves the haplotype reconstruction accuracy by taking genomic distances between SNVs into account. The observed decline in phasing information with increasing distance between SNVs is likely due to the formation of highly interacting genomic regions and organizational chromatin structures such as self-interacting TADs (Mb scale) and higher order metaTADs (Fraser *et al*., 2015; Razin *et al.*, 2016; Ulianov *et al.*, 2016). The MaxST obtained through this proximity-scaled weighted graph discards potential noise and assigns more importance to more likely co-observations of SNVs within neighbouring genomic regions. This runs the theoretical risk of breaking phasing blocks in situations where the only connecting variants were distant in genomic coordinates. In our analysis, no phasing blocks were broken due to proximity scaling of edge weights.

In summary, GAMIBHEAR enables accurate phasing of GAM data with average SERs ( ∼2%) comparable to those obtained with Hi-C (∼1.4%) (Chaisson *et al.*, 2019; Selvaraj *et al.*, 2013). While dedicated experimental techniques such as StrandSeq can yield dramatically lower SERs (Chaisson *et al.*, 2019), application of additional experimental techniques to resolve haplotypes more accurately is often not warranted or not feasible due to limited material or costs involved. While GAMIBHEAR is ultimately intended to be used on human data, no GAM dataset of sufficient size is yet available on human samples. In the meantime, the F123 cell line is well-suited to accurately measure phasing performance due to its known haplotype structure before adapting the algorithm to the characteristics of human genomes. Application of our proximity-scaled graph phasing algorithm on F123 GAM data downsampled to human SNV density suggests that the reconstruction of haplotypes is suitable and well applicable for the use in human data as well.

## 5 Conclusion

Understanding the effect of genetic variation on chromatin conformation and gene regulation is a key question in genomics research. Large consortia, such as the 4D Nucleome project (Dekker *et al.*, 2017), are now bundling resources to address open questions in this field and thus allele-specific analyses of chromatin conformation and other sources of genomic variation are moving increasingly into the spotlight (Cavalli *et al.*, 2019). The recently established GAM method (Beagrie *et al.*, 2017) offers a unique opportunity towards high-resolution allele-specific analyses of chromatin contacts in humans, and GAMIBHEAR provides the necessary algorithmic advances towards generating highly accurate, chromosome-spanning haplotypes from GAM data on human samples in the future.

## Supplementary Material

btab238_Supplementary_Data
