# Supplementary material for: GAMIBHEAR: whole-genome haplotype reconstruction from Genome Architecture Mapping data
Source: Bioinformatics. 2021 Apr 8;37(19):3128–35. doi: 10.1093/bioinformatics/btab238 (PMC8504635; doi:10.1093/bioinformatics/btab238)
Supplement: btab238_Supplementary_Data [file btab238_supplementary_data.zip › GAMIBHEAR_Supplementary_final_submission_bioinformatics_April2021.pdf]

Supplement

S1 Benchmark Genome

We use the hybrid mouse embryonic stem cell line F123 as a benchmark system for assessing the quality of reconstructed haplotypes from GAM data. The F123 line was derived from the F1 generation of two fully inbred homozygous mouse strains: *Mus musculus castaneus* (CAST) and *Mus musculus domesticus 129S4/SvJae* (J129) (Gribnau *et al.*, 2003).
In order to derive benchmark haplotypes of F123, whole-genome sequencing (WGS) data of CAST and J129 were downloaded from the European Nucleotide Archive (accession number ERP000042) and the Sequence Read Archive (accession number SRX037820), respectively. WGS reads were trimmed using Cutadapt (Martin, 2011) and mapped to the mouse reference genome mm10 using BWA (Li and Durbin, 2009). To determine the haplotypes of the F123 line, SNVs of both parental strains were identified using bcftools (Li, 2011) and SNVs covered by < 5 reads or quality < 30 were excluded.
With the haplotype structure thus known, this cell line serves as the benchmark for all downstream experiments and analyses.

S2 GAM Dataset, pre-processing and quality control

1281 individual GAM NuPs of the F123 line were generated from the F123 mESC cell line. The F123 SNVs were N-masked in the mm10 reference genome and reads were mapped using Bowtie2 (Langmead and Salzberg, 2012). Duplicate reads were removed using samtools (Li *et al.*, 2009). After mapping, all BAM files and WGS results underwent standard quality control using FastQC (Andrews, 2010) and multiQC (Ewels *et al.*, 2016). Reads were trimmed using BamUtil (Jun *et al.*, 2015) with function trimBam where necessary.
For quality assessment of each sample, the genome was split into fixed windows of size 50kb. For each NuP i and each window j, the number of reads r_{ij} and number of nucleotides covered c_{ij} were determined using bedtools (Quinlan and Hall, 2010). Windows were then classified as *positive* or *negative* based on r_{ij} and c_{ij} as follows: From the coverage $c_{i\cdot}$ of all windows for NuP i the empirical nucleotide coverage distribution P_i was computed. From P_i, the minimum coverage percentile MCP_i was chosen such that every window contains three or more reads. The average $\overline{MCP}$ across all NuPs then determined the sample-specific nucleotide coverage thresholds t_i (in bp) for each NuP. Windows w_{ij} were called positive iff $c_{ij} > t_i$, i.e. if the number of nucleotides covered in each window was greater than the sample-specific threshold and negative otherwise. *Positive* windows flanked by *negative* windows on each side were defined as *orphan* windows.
NuPs selected for further analysis had < 60% orphan windows and > 20,000 uniquely mapped reads. 1123 NuPs (89%) passed these quality thresholds (available under 4D Nucleome Consortium data portal accession number 4DNBSTO156AZ, unique 4DN identifiers in Supplementary Data).
Reads were then counted at known heterozygous SNV positions using samtools mpileup (Li *et al.*, 2009). Because of the frequently low coverage from independent (i.e. non-duplicate) reads at most positions (30% of observed SNVs are covered by 2 or fewer reads, 50% by 5 or fewer reads), we counted an allele as present if it was observed in at least one read at the examined position.

S3 Dataset Statistics

Benchmark genome (F123). The F123 mouse embryonic stem cell line was derived from a hybrid F1 mouse resulting from the cross of the two inbred, homozygous mouse strains CAST (*Mus musculus castaneus*) and J129 (*Mus musculus domesticus J129*). The parental mouse strains are both fully sequenced, their exclusively homozygous genomic variants with respect to the reference mouse genome mm10, which was derived from the mouse strain C57BL/6, are known. The F1 generation resulting from the cross of CAST and J129 is thus heterozygous at all loci for which their parents have different alleles. Their haplotypes are known, making them an ideal model for benchmarking phasing algorithms. Relative to the mouse reference genome mm10, CAST and J129 show 18,892,144 and 4,778,766 germline variants respectively, in concordance with their estimated evolutionary distance from C57BL/6, 371,000 ± 91,000 years (Goios *et al.*, 2007) and approximately 100 years (Simpson *et al.*, 1997), respectively. After exclusion of 2,200,819 overlapping SNV positions and 1,119,044 SNVs located in genomic regions of low mappability, the F123 reference set contains 18,150,228 variants in total, all of which are heterozygous due to inbreeding of the parental strains. Of those, 15,810,835 variants (87.1%) are located on the CAST parental genome, 2,339,393 (12.9%) on J129. This yields an average SNV density of 1 SNV per 132bp, with a median genomic distance of 56 bp.

Nuclear profiles. We obtained 1281 GAM NuPs of the F123 mESC cell line (4D Nucleome Consortium data portal accession number 4DNBSTO156AZ), out of which 1123 passed quality screening (see Supplementary Note S2).
We extracted on average 305,377 reads from each NuP, covering 0.171% (± 0.167) of the 18,150,228 heterozygous SNVs per nuclear slice (Figure 2A); exemplary data of genomic regions captured in a single NuP is shown in Figure 2B. Out of all F123 SNVs, 11,741,055 (64.69%) were observed at least once across all 1123 NuPs and 7,605,321 SNVs (41.9%) were observed at least twice (Figure 2C). Due to this sparsity and the fact that homologous chromosome pairs occupy distinct chromosomal territories (Khalil *et al.*, 2007), 96.54% of SNV observations showed counts from only one parental allele within one sample. Thus, we removed observed variants with read counts from both parental alleles without substantial loss of information. Since the slicing of nuclei in the GAM experiments is a random process, a balanced observation ratio of alternative and reference alleles of heterozygous SNVs is expected across all NuPs. We thus additionally removed all 550 variants (0.000045%) which significantly deviated from a balanced representation of reference and alternative alleles ($p < 0.05$ after Benjamini-Hochberg adjustment, binomial test against 0.5).

S4 Quality measures for reconstructed haplotypes

We here provide details about the employed measures of completeness and accuracy of the reconstructed haplotypes. The measures were chosen to allow comparison between the conceptually different neighbour and graph phasing algorithms and to allow comparison with existing methods. We calculate all SNV-based metrics per chromosome, relative to the number of phasable SNVs M_c on chromosome c, i.e. the number of heterozygous SNVs observed at least once in all 1123 NuPs. Analogously, we calculate all metrics based on genomic range (in bp) relative to the phasable genome per chromosome (distance between leftmost SNV and rightmost SNV in bp). We omit chromosome index c for brevity in the definitions below and report means and standard deviations of all measures across chromosomes in the Results section of the main text (Table 1). For the number of phasable SNVs and the size of the phasable

genome in bp see Supplementary Table 1 below. For a detailed discussion of GAM sparsity see Results and Discussion in the main text.

Supplementary Table 1: Description of the phasable SNV set and genome per chromosome. *Full SNV set* describes the phasable SNV set (M_c) and genome as observed in the F123 cell line. *Subsampled* corresponds to the phasable SNV set and genome after employing the downsampling strategy to mimic SNV density in the human genome (Results section 3.3.2).

Chr	Phasable SNV set (%)		Phasable genomic range in bp (%)		Chr	Phasable SNV set (%)		Phasable genomic range in bp (%)	
	Full SNV set	Sub-sampled	Full SNV set	Sub-sampled		Full SNV set	Sub-sampled	Full SNV set	Sub-sampled
chr1	941707 (63.08)	127839 (8.37)	192365707 (98.41)	192357993 (98.41)	chr11	640116 (68.96)	86657 (9.10)	118880794 (97.38)	118875736 (97.37)
chr2	782822 (66.01)	106135 (8.60)	178962141 (98.27)	178952453 (98.26)	chr12	533072 (62.68)	72156 (8.33)	117018721 (97.42)	116999097 (97.39)
chr3	694766 (59.03)	94652 (7.83)	156938923 (98.06)	156927079 (98.06)	chr13	588006 (64.44)	79840 (8.56)	117318662 (97.42)	117316342 (97.42)
chr4	741959 (66.33)	100655 (8.77)	153307607 (97.96)	153303916 (97.95)	chr14	553535 (63.08)	74866 (8.41)	121762587 (97.49)	121740416 (97.47)
chr5	738731 (64.94)	100240 (8.54)	148729017 (97.96)	148722202 (97.95)	chr15	494179 (63.75)	66861 (8.43)	100886142 (96.97)	100866521 (96.95)
chr6	726343 (63.99)	98125 (8.51)	146535902 (97.86)	146533104 (97.86)	chr16	455935 (60.53)	61556 (7.99)	95024075 (96.75)	94991322 (96.72)
chr7	687475 (67.40)	93734 (9.03)	142338158 (97.87)	142297146 (97.84)	chr17	431796 (65.11)	58301 (8.66)	91886960 (96.74)	91869956 (96.72)
chr8	688032 (67.27)	93486 (8.88)	126251015 (97.59)	126226500 (97.55)	chr18	474538 (65.43)	64371 (8.65)	87601865 (96.58)	87592598 (96.57)
chr9	599812 (66.89)	81359 (8.81)	121492381 (97.51)	121483172 (97.50)	chr19	303591 (68.90)	41486 (9.16)	58235870 (94.81)	58184777 (94.71)
chr10	664640 (64.07)	90115 (8.44)	127492655 (97.55)	127483223 (97.54)	**sum**	**11741055 (64.69)**	**1553527 (8.56)**	**2403029182 (97.58)**	**2402723553 (97.56)**

Completeness and contiguity measures

As a first measure of completeness we report the proportion of heterozygous SNVs and the proportion of neighbouring transitions that have been successfully phased. Because these measures do not take the contiguity of the phased blocks into account we additionally employ metrics that assess the size of the reconstructed haplotype blocks: the S50 (Lo *et al.*, 2011), N50 (Lander *et al.*, 2001) and AN50 (Lo *et al.*, 2011) metrics. A graphical explanation of the completeness measures S50, N50 and AN50 is shown in Supplementary Figure 1A.

Number of phased SNVs / transitions

The absolute number of phased SNVs m_{phased} and its relative counterpart $p_{phased} = m_{phased} / M$ give a general overview of phasing completeness. In the case where phasing

yields a large number K of small, disconnected haplotype blocks, the number of phased SNVs will be high, but the phase between these independent blocks is unknown. To account for this fragmentation, we report t_{phased} , the frequency of phased transitions between adjacent SNVs. As the number of transitions is equal to the number of SNVs $M-1$ and each additional block beyond the first incurs one unphased transition, this yields:
$t_{phased} = (M-1) - (K-1) = M-K.$

S50

S50 (Lo *et al.*, 2011) is a measure of contiguity, i.e. of the size distribution of phased haplotype blocks. To obtain the S50 value, all phased haplotype blocks are sorted by their size (number of SNVs phased in the block), and the S50 value is the size of the block at which 50% or more of SNVs are phased. For example, an S50 value of 1000 SNVs would mean that 50% of all SNVs are contained in haplotype blocks of size 1000 SNVs or larger.

N50 / AN50

Analogously, to obtain the N50 contiguity metric (Lander *et al.*, 2001), the phased haplotype blocks are sorted by their genomic span (in bp) to determine the span at which 50% or more of the phasable genome is phased. To correct for cases where isolated haplotype blocks are contained within larger blocks spanning them (see Supplementary Figure 1A), we also report the adjusted N50 (AN50, (Lo *et al.*, 2011)), where the genomic span of the block is adjusted by the fraction of SNVs phased within. Since haplotype blocks reconstructed by the neighbour phasing approach are never nested (Supplementary Figure 1B), N50 = AN50 for neighbour phasing.

Accuracy measures

To assess the accuracy of the reconstructed haplotypes we compare GAMIBHEAR estimates with the haplotypes of the F123 mouse embryonic stem cell (mESC) line obtained from whole-genome sequencing of the parental mouse strains (see Supplementary Note S1 'Benchmark genome (F123)'). Two measures are considered: the global haplotype agreement calculated by direct comparison of the reconstructed and true haplotypes (i.e. alt-ref configurations) as a global measure of accuracy, and the Switch Error Rate (SER) as a local measure of accuracy (see also Supplementary Figure 1B).

Global haplotype accuracy

We report as global haplotype accuracy the overall agreement between haplotypes assigned to SNVs after phasing and their true assignment (see Supplementary Note S1). Let $\hat{h} \in \{-1,1\}^M$ be the inferred haplotype assigned to SNV at position i and let $h \in \{-1,1\}^M$ be the true haplotype assignment. The phasing error e is then defined as:

$$e = \frac{1}{M}\sum_{i=1}^{M}\frac{1}{2}|h_i - \hat{h}_i|$$

Since the true parent of origin of a variant cannot be identified, haplotypes are equivalent to their full complement in terms of phasing accuracy (for example $h = (-1, 1, -1)$ is equivalent to $h' = (1, -1, 1)$). Global accuracy can thus never drop below 50% and the final global haplotype accuracy G is thus:

$$G = max(e, 1-e)$$

As the first SNV of every haplotype block is arbitrarily set to $h_1 = 1$, global accuracy for phasing results with many blocks is highly sensitive to the true distribution of alternative alleles over the parental haplotypes. In the F123 dataset, 87 % of alternative alleles reside on the CAST haplotype. A naive phasing algorithm, which places all alternative alleles on haplotype 1 would thus yield a global accuracy of $G = 0.87$. This is visible in the seemingly high $G = 0.86$ of the neighbour phasing algorithm despite its low completeness and contiguity.

Switch Error Rate (SER)

We report the Switch Error Rate (SER, Supplementary Figure 1B) as a local accuracy metric. Analogous to the global haplotype accuracy, the SER is defined as the proportion of adjacent variant pairs that were phased incorrectly out of all phased variant pairs. For each haplotype block $k \in \{1,..,K\}$ we transform the inferred and true haplotype vectors $\hat{h}(k) \in \{-1,1\}^{M_k}$ and $h(k) \in \{-1,1\}^{M_k}$ into the inferred vector $\hat{t}(k) \in \{-1,1\}^{M_k-1}$ and true transition vector $t(k) \in \{-1,1\}^{M_k-1}$, where 1 and -1 correspond to *stay* and *flip* transitions, respectively. The SER across all haplotype blocks k is thus

$$SER = \frac{1}{M-K}\sum_{k=1}^{K}\sum_{i=1}^{M_k-1} |t(k)_i - \hat{t}(k)_i|$$

, with: $M = \sum_{k=1}^{K} M_k$ (total number of phased SNVs).
The factor $1/(M-K)$ makes the SER relative to all phased transitions.

Adjusted SER

Transitions without phasing information that are arbitrarily assigned a *stay* or *flip* state have a 50% chance of being correct, irrespective of the true distribution of alternative alleles over the parental haplotypes. To account for this, we add a SER penalty of 0.5 per unphased transition to define the adjusted SER:

$$SER_{adj} = \frac{1}{M-1}(0.5(K-1) + \sum_{k=1}^{K}\sum_{i=1}^{M_k-1} |t(k)_i - \hat{t}(k)_i|)$$

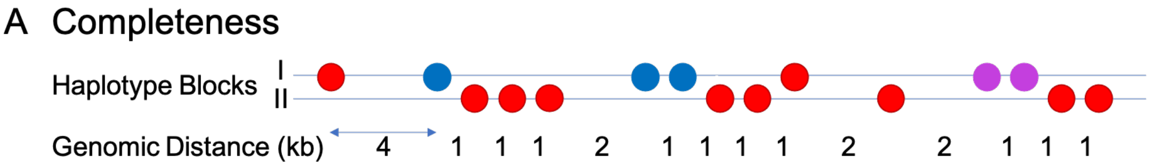

	●	●	●
Size	10 SNV (66.6 %) (S50)	3 SNV (20 %)	2 SNV (13.3 %)
Span	20 kb (100 %) (N50)	6 kb (30 %)	1 kb (5 %)
Adjusted Span	20 kb * 10/15 = 13.3 kb (66 %) (AN50)	6 kb * 3/6 = 3 kb (15 %)	1 kb (5 %)

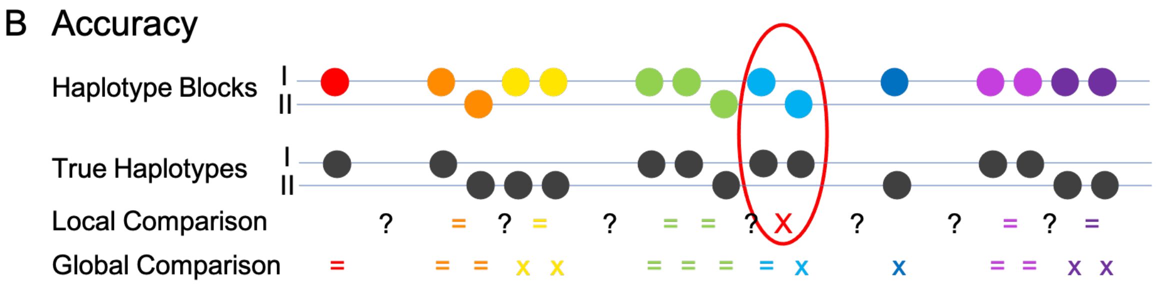

$$SER = \frac{1\ incorrectly\ phased\ transition}{7\ phased\ transitions} = 14.3\ \%$$

$$Adjusted\ SER = \frac{1\ incorrectly\ phased\ transition + (0.5 \cdot 7)\ unphased\ transitions}{14\ phasable\ transitions} = 32.1\ \%$$

$$Global\ accuracy = \frac{9}{15}\ haplotype\ agreements = 60\ \%$$

Supplementary Figure 1: Graphical explanation of quality measures A) Completeness: a schematic graph phasing result is shown, consisting of 3 nested haplotype blocks (red, blue, purple). Size (number of SNVs), genomic span and genomic span adjusted by the fraction of phased SNVs within are exemplarily calculated for the 3 blocks respectively. When ordering the blocks by size, the red block contains more than 50% of the SNV set and thus its size corresponds to the reported S50, N50, AN50 respectively. **B) Accuracy:** a schematic neighbour phasing result is shown, consisting of multiple non-overlapping haplotype blocks. Switch Error Rate (SER) is calculated for the presented haplotype reconstruction, one out of 7 phased transitions is incorrect (circled, marked with a red X). The SER is then adjusted by unphased transitions (marked as ?), which are penalized by 0.5 switch errors. Global Comparison of assignments of alternative alleles to parental haplotypes shows 9 concordant (=) and 6 dissenting (x) assignments, resulting in a global accuracy of 60%.

S5 Reconstruction accuracy per chromosome

Here we report global and local accuracy (SER) results of haplotypes reconstructed using the neighbour phasing, basic and proximity scaled graph phasing algorithms per chromosome in Supplementary Table 2. In general, the global accuracy of reconstructed haplotypes improves with the complexity of the used algorithms. Noticeably, the SER metric shows a smaller range in results over chromosomes compared to the global accuracy, which shows outliers. In general, SER is a more meaningful metric compared to global haplotype accuracy, where a single switch error in a haplotype block can lead to the following part of the haplotype block being assigned to the opposite haplotype, thus drastically decreasing global accuracy while maintaining high

local accuracy. Supplementary Figure 2 shows one such example of the high impact of switch errors on global haplotype accuracy on an outlier result on chromosome 17.

Supplementary Table 2: Global Accuracy (GA) and switch error rate (SER) per chromosome. Haplotypes were reconstructed from the full data set of 1123 GAM NuPs using the neighbour phasing, graph phasing and scaled graph phasing approach. Final haplotype assignments, independent of haplotype blocks, are compared with the known F123 haplotypes. Percent of concordant haplotype assignments (GA, higher is better) and switch error rates of phased transitions (SER, lower is better) are shown per chromosome, as well as mean, median and standard deviation over chromosomes.

Chr	Neighbour phasing		Graph phasing		Scaled graph phasing	
	GA	SER	GA	SER	GA	SER
chr1	85.51	0.71	95.14	5.31	98.03	1.98
chr2	87.22	0.74	94.92	5.80	98.00	2.08
chr3	87.69	0.70	94.16	6.22	97.64	2.20
chr4	84.55	0.99	94.19	6.40	97.32	2.49
chr5	88.29	0.74	95.44	5.12	87.44	2.03
chr6	87.50	0.67	95.93	4.64	98.08	1.89
chr7	85.26	0.84	95.75	5.01	97.99	2.13
chr8	79.99	0.93	95.44	5.28	84.51	2.29
chr9	84.85	0.83	94.94	5.90	97.78	2.25
chr10	93.56	0.51	95.87	4.56	98.45	1.53
chr11	88.90	0.68	95.20	5.49	98.14	1.87
chr12	85.50	0.81	94.85	5.60	85.69	2.25
chr13	87.98	0.63	95.409	5.07	98.11	1.88
chr14	78.16	0.92	95.505	5.12	97.65	2.33
chr15	81.04	0.88	93.85	6.11	97.58	2.36
chr16	88.71	0.54	95.42	5.01	98.14	1.76
chr17	83.718	0.93	95.16	5.60	64.79	2.54
chr18	85.19	0.74	95.39	5.22	97.96	1.98
chr19	87.84	0.68	94.92	5.51	97.99	1.93
**mean**	**85.87**	**0.76**	**95.13**	**5.42**	**94.28**	**2.09**
**median**	**85.51**	**0.74**	**95.20**	**5.31**	**97.96**	**2.08**
**sd**	**3.53**	**0.13**	**0.57**	**0.50**	**8.45**	**0.26**

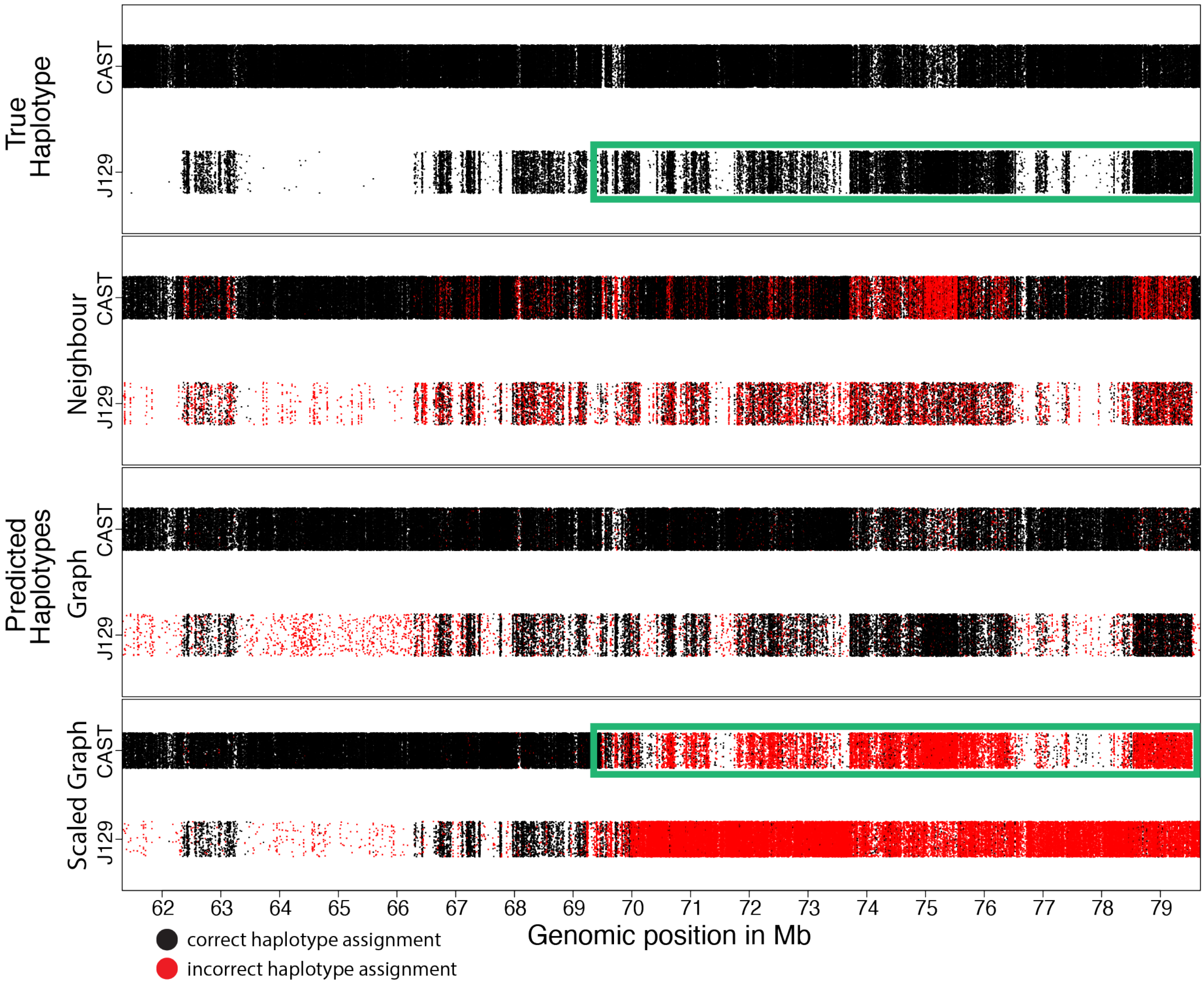

Supplementary Figure 2: Outlier of decreased global accuracy caused by switch error on chromosome 17. The 4 panels show the location of alternative alleles of heterozygous SNVs along the parental chromosome copies CAST (upper band) and J129 (lower band) in a genomic region on chromosome 17 (61Mb - 80Mb). Each dot represents one SNV, the majority of SNVs is located on the CAST chromosome copies. Panel 1 shows the true haplotypes, meaning the true location of alternative alleles on the parental chromosome copies. Panels 2-4 show the predicted haplotype assignments reconstructed from neighbour phasing (panel 2), graph phasing (panel 3) and scaled graph phasing (panel 4). Black dots represent correct assignments, red dots show incorrect assignments of haplotypes. Graph phasing shows a clear improvement in global accuracy compared to neighbour phasing results. The last panel shows the impact of switch errors within haplotype blocks on the global accuracy: a switch error between 69Mb and 70Mb causes the subsequent haplotype assignment to switch onto the opposite haplotype, causing reduced global accuracy while maintaining local accuracy. The pattern formed by the distribution of J129 SNVs (as shown in panel 1) is still clearly visible in panel 4 after the switch error, only incorrectly predicted to be located on the CAST chromosome copy (highlighted in green boxes), thus demonstrating that the local phasing prediction is still highly accurate.

S6 Effect of window size in graph phasing approach

Unless indicated otherwise, all reported results are haplotype reconstructions using default parameter settings. In the graph phasing approach haplotypes are not reconstructed chromosome wide, but in overlapping windows of (default 20,000) SNVs in order to ensure successful completion of calculations without exceeding time and memory limits. We tested

the impact of changing window sizes on the quality of reconstructed haplotypes as well as time and memory usage from a minimum of 10,000 SNVs to a maximum of 40,000 SNVs per window. Reducing or increasing the window size only marginally affected the performance of the algorithm in terms of completeness or accuracy; however, it did show a definite impact on the runtime and memory usage (See Supplementary Table 3. Thus, changes to the default parameters should be made with care under consideration of local memory capacity.

Supplementary Table 3: Comparison of different window sizes L. Concerning runtime, memory consumption of the algorithm, as well as completeness and accuracy of reconstructed haplotypes. Scaled graph phasing was used to reconstruct haplotypes from the full dataset.

Metric	L = 10.000 SNVs	L = 20.000 SNVs	L = 30.000 SNVs	L = 40.000 SNVs
Runtime (elapsed = wall clock time)	02:50 h	05:09 h	07:55 h	10:31 h
Memory consumption	20 GB	30 GB	62 GB	106 GB
Mean number of blocks	119	76	57	46
% SNVs phased in largest block	99.90 %	99.94 %	99.95 %	99.96 %
Global Accuracy	94.29 %	94.28 %	94.28 %	94.27 %
SER	2.07 %	2.09 %	2.10 %	2.11 %

S7 Lower SNV density

The F123 mESC cell line has a relatively high SNV density (8 SNVs per 1kb) compared to humans (approximately 1-1.5 SNVs per 1kb, (1000 Genomes Project Consortium *et al.*, 2015)). To show the effect of SNV density on the quality of haplotype reconstructions, we randomly subsampled the F123 SNV set to resemble human SNV density and evaluated the resulting haplotypes. In order to obtain an average SNV density of 1 SNV per 1kb, we retained 2,462,745 (13.57%) out of the known 18,150,228 F123 SNVs in the 2.46 billion bp mm10 mouse reference genome. The distribution of SNVs along the parental chromosomes remained constant (full SNV set: 87.11% CAST, 12.89% J129; subsampled: 87.14% CAST, 12.86% J129). Variants were randomly subsampled from the true parental haplotypes irrespective of their observation in the GAM NuPs. Similar to the full dataset (64.69% of known SNVs observed), 64.66% of all SNVs were observed in the subsampled dataset.

We explored accuracy and completeness of the best-performing proximity-scaled graph phasing algorithm on the subsampled dataset. All parameters, including the proximity scaling parameters, remained unchanged for the haplotype reconstruction. Despite the reduced SNV density and thus increased genomic distance between co-observed SNVs, GAMIBHEAR reconstructed accurate, dense, chromosome-spanning haplotypes: 99.96% of input SNVs were phased into haplotype blocks of minimum size 2, on average 99.95% (± 0.0096%) of those were phased in the main, chromosome-spanning haplotype block, covering 100% (± 0.00%) of the phasable genome.

The mean global accuracy of 87.46% is still fairly high, the high standard deviation of ± 15.21% indicates a large span in the results. The median global accuracy of 96.64% and the switch error rate of 4.84% (± 0.6%) show that the quality of the reconstructed haplotypes in a subsampled dataset is only slightly different from that of the haplotypes reconstructed from the full dataset, indicating that the algorithmic approach is largely independent of SNV density and thus applicable to human data.

S8 Comparison with MEC solvers WhatsHap and HapCHAT

GAMIBHEAR is the first algorithm specialized in the usage of GAM data for haplotype reconstruction. GAM data stores phasing information differently than Hi-C data or PacBio long reads, which are frequently used for haplotype reconstruction with existing phasing algorithms such as HapCUT2 (Edge *et al.*, 2017), WhatsHap (Patterson *et al.*, 2015), HapCol (Pirola *et al.*, 2016) and HapCHAT (Beretta *et al.*, 2018).

Chimeric reads from Hi-C experiments store phasing information if both chimeric parts overlap at least one SNV each. If this is the case, phasing information between these two genomic regions is captured, as intrachromosomal contacts are more likely than interchromosomal contacts. On the other hand, reads generated by the PacBio platform capture phasing information regarding all SNVs covered by one individual long read. When it comes to reads generated in GAM experiments, the phasing information is not stored within individual reads as it is the case with chimeric Hi-C reads or long reads, but the SNVs covered by all reads captured within one nuclear profile (NuP) convey accurate local phasing information.

In order to explore if the spatial phasing information from GAM data could be readily transformed for the use in existing phasing algorithms, we decided to transform GAM data into pseudo long reads, since reads in GAM NuPs are sequenced from strands of DNA captured in physical nuclear slices. Thus, all SNVs co-observed within one GAM NuP were treated as if they were all covered on one continuous long read.

For the following comparison we concentrated exclusively on chromosome 1 of the F123 GAM dataset. It contains 1,584,837 known heterozygous SNVs, of which 941,707 are observed in 1110 GAM NuPs. 1087 NuPs contain at least 2 observed SNVs, which is the minimum number of SNVs necessary to convey phasing information. These NuPs were transformed into 1087 pseudo long reads, each read covering between 2 and 25,776 SNVs (on average 2,486 SNVs). Similar to the ternary $N \times M$ matrix D described in section 2.1, D' was created from the 1087 pseudo long reads covering 941,704 SNVs in total. D' was built to meet the tools' internal representation of reference, alternative and not observed alleles as $\{0, 1, -\}$, respectively, and then used as direct input to the wMEC and k-constrained MEC solvers WhatsHap and HapCHAT, using default parameters.

WhatsHap is fixed parameter tractable in the coverage and sets a default coverage threshold of 15x (maximum 23x) since PacBio long read data is characterized by uniform read and SNV coverage. However, unlike in PacBio long read data, SNV coverage in the sparse GAM data is usually low (chr1: on average 2.87x) but not uniform and varies, up to 37x on chr 1.

Thus, a few SNVs in D' (0.23%) exceed the default coverage of 15x, and 0.0073% exceed even the maximum coverage threshold (23x).

To ensure the compliance of its coverage threshold, WhatsHap uses a read selection heuristic to select suitable reads that are most informative for phasing. The read selection process of WhatsHap resulted in a loss of the majority of long reads, 69 reads (6.35%) remained. As the coverage of GAM data is usually low and most SNVs are only observed once, this stringent read selection resulted in a loss of the majority of considered SNVs. 11,039 SNVs (1.17% of input SNVs) were retained for subsequent phasing, but haplotypes containing approximately 1% of input SNVs would not be useful. In conclusion, as WhatsHap's read selection heuristic was designed for data sets where SNV coverage is uniform, GAM data cannot be readily transformed for its use in WhatsHap as it does not meet coverage requirements.

HapCHAT, based on WhatsHap and HapCol, was precisely developed to allow the consideration of datasets composed of higher coverages, as well as to improve the accuracy of computed haplotypes. In a preprocessing step, reads that are likely to originate from the same chromosome copy are merged. It was shown that, using read merging, HapCHAT can effectively handle datasets with approximately 60x coverage.

In our comparison, the 1087 pseudo long reads were merged into 691 reads. To fulfil the default coverage threshold of 15x, merged reads were downsampled using the same selection process as employed by WhatsHap. The 63 remaining merged pseudo long reads covered 604,358 SNVs (64.18% of input SNVs), all of which were subsequently phased into 5 haplotype blocks of minimum size 2. The largest block contained 604,350 SNVs (S50, 64.18%) and spanned 192,334,685 bp (99.993% of the phasable genomic range), creating a chromosome-spanning haplotype block. Adjusting its span for the fraction of phased SNVs yields 123,434,304 bp (AN50), which is equivalent to 64.17% of the phasable genomic range.

The haplotypes reconstructed by HapCHAT, which eventually phased 64.18 % of input SNVs show a global accuracy of 81.36%, with a SER of 11.38%. The MEC cost was reported as 307,734. A side-by-side comparison is shown in Supplementary Table 4.

Supplementary Table 4: Side-by-side comparison of HapCHAT and GAMIBHEAR phasing results on F123 chromosome 1.

Metric	HapCHAT		GAMIBHEAR	
	Absolute	Percent	Absolute	Percent
Phased variants	604,358 SNVs	64.18 %	941,400 SNVs	99.97 %
Number of Blocks	5	-	125	-
S50	604,350 SNVs	64.18 %	941,060 SNVs	99.93 %
N50	192,334,685 bp	99.99 %	192,348,818 bp	100 %
AN50	123,434,304 bp	64.17 %	192,216,665 bp	99.93 %
Global Accuracy	-	81.36 %	-	98.03 %
SER	-	11.38 %	-	1.98 %

We were curious on how these results would improve by increasing the default coverage threshold to the maximum possible coverage of 23x. While 2,187 SNVs (0.23%) of chromosome 1 show a higher coverage than 15x, only 69 SNVs (0.0073%) show a higher coverage than 23x. Unfortunately, while providing 400G of memory and no time limit, we were not able to finish the computation of results, as after approximately 30h the computation was aborted due to the excess of the memory limit. We believe that the high number of SNVs was responsible for this situation. Due to the high SNV density of F123 compared to human data, the set of 941,704 heterozygous SNVs on chromosome 1 is 19.6 fold larger than one of the benchmark SNV sets used in the HapCHAT paper. There a set of 48,023 heterozygous SNV on chromosome 1 of the individual NA24385, with a coverage of 60x, was phased in 1.5 h using 3.9 GB RAM, thus not exceeding the 64G memory and 24h runtime limits.

S9 Comparison with HaploSeq

In 2013 Selvaraj *et al.* presented HaploSeq, a method that combines Hi-C on the experimental side, and HapCUT on the algorithmic side, to reconstruct accurate haplotypes genome-wide.

HaploSeq, as well as GAMIBHEAR, were developed and validated on the F123 mESC line, which makes their results comparable. The authors of HaploSeq present quality metrics with respect to the largest haplotype block, defined as the block with the most variants phased (MVP block). To enable direct comparison between the approaches, we report here the same metrics as reported by Selvaraj *et al* (see Supplementary Table 5). Since GAMIBHEAR was developed to enable haplotype-specific analysis of GAM data without the need of further experiments, we primarily report our results with respect to the set of SNVs observed in the GAM data set, but additionally with respect to the full set of known heterozygous SNVs in F123.
Selvaraj *et al.*, 2013 report > 99.9% of each phasable chromosome spanned by the MVP block, phasing about 95% of variants into the largest block, and > 99.5% accurately phased SNVs.

Supplementary Table 5: GAMIBHEAR metrics comparable to HaploSeq.

Chr	Phasable span of chr		Variants spanned in MVP block		% chr spanned in MVP block		% variants phased in MVP block		% accuracy in MVP block
	observed	all	observed	all	observed	all	observed	all	same
chr1	192348817	192365707	941707	1492978	100	100	99.93	63.03	98.04
chr2	178961691	178962141	782822	1185899	100	100	99.94	65.97	98.01
chr3	156935404	156938923	694766	1177038	100	100	99.91	58.97	97.65
chr4	153307607	153307607	741959	1118621	100	100	99.94	66.29	97.33
chr5	148722916	148729017	738730	1137578	100	100	99.94	64.90	87.44
chr6	146535718	146535902	726343	1135114	100	100	99.94	63.95	98.09
chr7	142338158	142338158	687475	1019994	100	100.	99.95	67.37	98.00
chr8	126223967	126251015	688032	1022728	100	99.98	99.95	67.24	84.51
chr9	121472177	121492381	599812	896751	100	99.98	99.94	66.85	97.78
chr10	127492655	127492655	664640	1037319	100	100	99.94	64.03	98.48
chr11	118880794	118880794	640116	928226	100	100	99.95	68.93	98.15
chr12	117017998	117018721	533072	850430	100	100	99.92	62.63	85.71
chr13	117317721	117318662	588006	912425	100	100	99.93	64.40	98.15
chr14	121475018	121762587	553535	877497	100	99.76	99.94	63.04	97.68
chr15	100886010	100886142	494179	775168	100	100	99.94	63.71	97.60
chr16	94993997	95024075	455935	753275	100	99.97	99.93	60.48	98.15
chr17	91886804	91886960	431796	663159	100	100	99.94	65.07	64.79
chr18	87601477	87601865	474538	725320	100	100	99.94	65.38	97.99
chr19	58234352	58235870	303591	440643	100	100	99.95	68.86	97.99

When downsampling the F123 SNV set to human SNV density, Selvaraj *et al.* still report complete (>99.2 % of the phasable chromosomes spanned) and only marginally less accurate

(> 98.9%) MVPs, which, however, show a drastically lower resolution as the MVP blocks only phased approximately 32 % of SNVs.

In contrast, GAMIBHEAR was able to phase 99.96% of downsampled input SNVs, of which 99.95% are contained within the main, chromosome-spanning haplotype block. This block spans 100% of the phasable genome (97.56 % of the full genome) with a comparable global accuracy of 96.64%.

References

1000 Genomes Project Consortium *et al.* (2015) A global reference for human genetic variation. *Nature*, **526**, 68–74.

Andrews,S. (2010) FastQC - A quality control tool for high throughput sequence data. *Babraham Bioinformatics*.

Beretta,S. *et al.* (2018) HapCHAT: adaptive haplotype assembly for efficiently leveraging high coverage in long reads. *BMC Bioinformatics*, **19**, 252.

Edge,P. *et al.* (2017) HapCUT2: robust and accurate haplotype assembly for diverse sequencing technologies. *Genome Res.*, **27**, 801–812.

Ewels,P. *et al.* (2016) MultiQC: summarize analysis results for multiple tools and samples in a single report. *Bioinformatics*, **32**, 3047–3048.

Goios,A. *et al.* (2007) mtDNA phylogeny and evolution of laboratory mouse strains. *Genome Res.*, **17**, 293–298.

Gribnau,J. *et al.* (2003) Asynchronous replication timing of imprinted loci is independent of DNA methylation, but consistent with differential subnuclear localization. *Genes Dev.*, **17**, 759–773.

Jun,G. *et al.* (2015) An efficient and scalable analysis framework for variant extraction and refinement from population-scale DNA sequence data. *Genome Res.*, **25**, 918–925.

Khalil,A. *et al.* (2007) Chromosome territories have a highly nonspherical morphology and nonrandom positioning. *Chromosome Res.*, **15**, 899–916.

Lander,E.S. *et al.* (2001) Initial sequencing and analysis of the human genome. *Nature*, **409**, 860–921.

Langmead,B. and Salzberg,S.L. (2012) Fast gapped-read alignment with Bowtie 2. *Nat. Methods*, **9**, 357–359.

Li,H. (2011) A statistical framework for SNP calling, mutation discovery, association mapping and population genetical parameter estimation from sequencing data. *Bioinformatics*, **27**, 2987–2993.

Li,H. *et al.* (2009) The Sequence Alignment/Map format and SAMtools. *Bioinformatics*, **25**, 2078–2079.

Li,H. and Durbin,R. (2009) Fast and accurate short read alignment with Burrows-Wheeler transform. *Bioinformatics*, **25**, 1754–1760.

Lo,C. *et al.* (2011) Strobe sequence design for haplotype assembly. *BMC Bioinformatics*, **12 Suppl 1**, S24.

Martin,M. (2011) Cutadapt removes adapter sequences from high-throughput sequencing reads. *EMBnet.journal*, **17**, 10–12.

Patterson,M. *et al.* (2015) WhatsHap: Weighted Haplotype Assembly for Future-Generation Sequencing Reads. *J. Comput. Biol.*, **22**, 498–509.

Pirola,Y. *et al.* (2016) HapCol: accurate and memory-efficient haplotype assembly from long reads. *Bioinformatics*, **32**, 1610–1617.

Quinlan,A.R. and Hall,I.M. (2010) BEDTools: a flexible suite of utilities for comparing genomic features. *Bioinformatics*, **26**, 841–842.

Selvaraj,S. *et al.* (2013) Whole-genome haplotype reconstruction using proximity-ligation and shotgun sequencing. *Nat. Biotechnol.*, **31**, 1111–1118.

Simpson,E.M. *et al.* (1997) Genetic variation among 129 substrains and its importance for targeted mutagenesis in mice. *Nat. Genet.*, **16**, 19–27.
